# Geranylgeraniol Suppresses the Expression of IRAK1 and TRAF6 to Inhibit NFκB Activation in Lipopolysaccharide-Induced Inflammatory Responses in Human Macrophage-Like Cells

**DOI:** 10.3390/ijms20092320

**Published:** 2019-05-10

**Authors:** Puspo E. Giriwono, Hitoshi Shirakawa, Yusuke Ohsaki, Shoko Sato, Yukihide Aoyama, Hsin-Jung Ho, Tomoko Goto, Michio Komai

**Affiliations:** 1 Laboratory of Nutrition, Graduate School of Agricultural Science, Tohoku University, 468-1 Aramaki Aza Aoba, Aoba-ku, Sendai 980-8572, Japan; pegiriwono@ipb.ac.id (P.E.G.); ohsaki@med.tohoku.ac.jp (Y.O.); satosho@iam.u-tokyo.ac.jp (S.S.); y.aoyama@g-mail.tohoku-university.jp (Y.A.); kinyou@g-mail.tohoku-university.jp (H.-J.H.); tgoto@mgu.ac.jp (T.G.); mkomai@m.tohoku.ac.jp (M.K.); 2 Southeast Asian Food & Agriculture Science & Technology (SEAFAST) Center, Bogor Agricultural University, Bogor 16680, Indonesia; 3 International Education and Research Center for Food Agricultural Immunology, Graduate School of Agricultural Science, Tohoku University, Sendai 980-8572, Japan

**Keywords:** anti-inflammation, geranylgeraniol, IRAK1, isoprenoid, TRAF6

## Abstract

Geranylgeraniol (GGOH), a natural isoprenoid found in plants, has anti-inflammatory effects via inhibiting the activation of nuclear factor-kappa B (NFκB). However, its detailed mechanism has not yet been elucidated. Recent studies have revealed that isoprenoids can modulate signaling molecules in innate immune responses. We found that GGOH decreased the expression of lipopolysaccharide (LPS)-induced inflammatory genes in human macrophage-like THP-1 cells. Furthermore, we observed that the suppression of NFκB signaling proteins, in particular interleukin-1 receptor-associated kinase 1 (IRAK1) and tumor necrosis factor receptor-associated factor 6 (TRAF6), occurred in GGOH-treated cells prior to LPS stimulation, suggesting an immunomodulatory effect. These results indicate that GGOH may modulate and help prevent excessive NFκB activation that can lead to numerous diseases.

## 1. Introduction

Nuclear factor-kappa B (NFκB) is a stress-responsive transcription factor involved in various cellular events, including cell proliferation, differentiation, carcinogenesis, autophagy, neurodegeneration, and acute and chronic inflammation [1,2,3,4,5,6,7,8,9]. The activation of NFκB occurs via distinct signal transduction pathways by extracellular stimuli and intracellular transduction pathways. Activated NFκB in the cytoplasm then translocates into the nucleus and stimulates the expression of its numerous target genes. The enhancement of mRNA expression of NFκB target genes is physiologically necessary as a response to various types of stress; however, excessive, continuous expression of these mRNAs is associated with several diseases. Especially, the activation of NFκB, mediated by toll-like receptor 4 (TLR4), is involved not only in an acute inflammatory response, but also in the chronic, low-grade inflammation that can induce metabolic syndrome [10]. Thus, the inactivation and suppression of NFκB are thought to be crucial for the prevention and alleviation of related diseases [11]. Numerous papers have indicated that plants produce effective secondary metabolites for the inactivation and suppression of TLR4-mediated NFκB activation [12]. Such ingredients are contained in our daily consumed foods. For example, epigallocatechin-3-gallate in green tea can inhibit the TLR4–NFκB signal transduction axis [13].

Geranylgeraniol (GGOH), a natural C20 isoprenoid found in plants and structurally similar to the side chain of menaquinone-4 (one form of vitamin K_2_), exhibited anti-inflammatory actions in human peripheral blood mononuclear cells (PBMC) [14,15], as well as in a mouse model of alendronate-induced inflammation [16]. GGOH also maintained endotoxin tolerance in murine peritoneal macrophages [17] and suppressed the expression of lipopolysaccharide (LPS)-induced inflammatory cytokines in bisphosphonate-treated RAW264.7 cells [18,19]. Furthermore, it has been demonstrated that GGOH, in addition to other isoprenoids such as β-ionone and ursolic acid, inhibited NFκB activation [20,21]. We previously showed that the ingestion of GGOH suppressed LPS-induced inflammation in rats [22]. In addition, GGOH suppressed LPS-induced proinflammatory cytokine expression in human macrophagic THP-1 cells [23]. However, the detailed mechanism of its anti-inflammatory action has not been elucidated. The aim of this study was to analyze how GGOH suppresses LPS-induced proinflammatory cytokine expression in differentiated THP-1 cells. We observed that GGOH plays a role in suppressing signal transducers, particularly interleukin-1 receptor-associated kinase 1 (IRAK1) and tumor necrosis factor receptor-associated factor 6 (TRAF6), to substantially inhibit NFκB activation.

## 2. Results

### 2.1. GGOH Suppresses LPS-Induced Inflammatory Genes in Macrophagic THP-1 Cells

**Figure 1:**
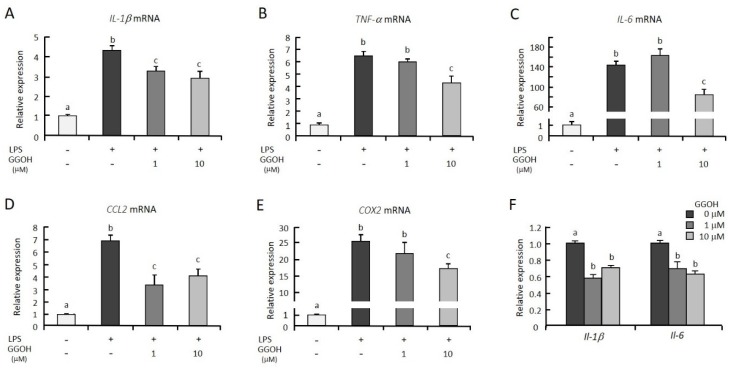
Geranylgeraniol (GGOH) suppressed the mRNA levels of inflammatory genes in lipopolysaccharide (LPS)-stimulated THP-1 and RAW264.7 cells. (**A**–**E**) THP-1 cells were differentiated with phorbol 12-myristate 13-acetate (PMA), then preincubated with or without GGOH for 24 h. After a 3 h LPS stimulation, the cells were harvested, and the RNA was isolated, as described in the experimental procedures. (**F**) Murine RAW264.7 cells were preincubated with GGOH for 24 h. After a 3 h stimulation with LPS, the cells were harvested, and the RNA was isolated. The mRNA levels of the inflammatory genes were then measured by quantitative RT-PCR. All values represent the mean ± SEM; *n* = 3. The values with different letters (a, b, and c) are significantly different at *p* < 0.05, as assessed by one-way analysis of variance, followed by Fisher’s least significant difference test.

Previously, we observed that the pretreatment of GGOH for 24 h suppressed LPS-induced interleukin-6 (IL-6) expression in differentiated THP-1 cells [23] Thus, in this study, we first examined whether GGOH treatments suppressed the expression of other proinflammatory genes induced by LPS in macrophagic THP-1 cells. The mRNA levels of the inflammatory cytokines interleukin-1β (IL-1β), tumor necrosis factor-α (TNF-α), and IL-6 were markedly increased 3 h after LPS treatment (Figure 1A–C). The cells pretreated with GGOH (10 μM) for 24 h before LPS stimulation showed significant suppression of the mRNA levels of these genes. However, we did not see effects on cell viability with GGOH treatment up to 10 μM (Figure S1). These results are consistent with our previous results [23]. Further, GGOH decreased the mRNA levels of C-C motif chemokine ligand 2 (CCL2) and cyclooxygenase-2 (COX2), which are regulated by NFκB (Figure 1D,E). However, treatment with GGOH simultaneously with LPS stimulation did not show suppressive effects on the expression of the proinflammatory cytokines. Pretreatment of murine macrophagic RAW264.7 cells with GGOH also suppressed LPS-induced Il-1β and Il-6 mRNA upregulation (Figure 1F). These results indicated that GGOH pretreatment suppressed LPS-induced inflammatory gene expression.

### 2.2. GGOH Suppresses NFκB Activation

**Figure 2:**
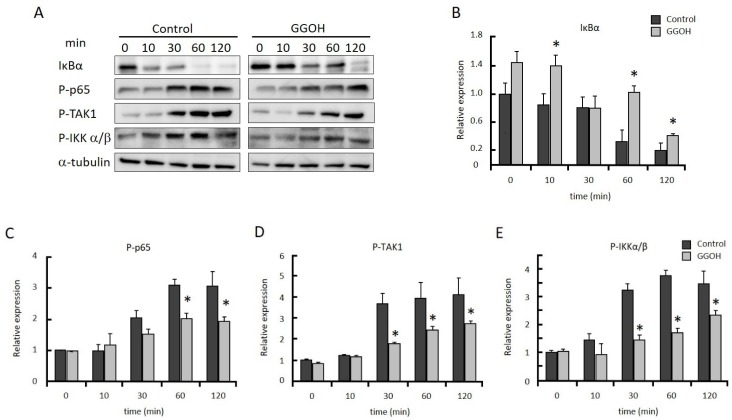
GGOH prevents the activation of NFκB in LPS-stimulated THP-1 cells. Differentiated THP-1 cells were preincubated with or without GGOH for 24 h before LPS stimulation. The cells were harvested after the indicated time of LPS treatment and the protein levels were measured by western blot, as described in the experimental procedures (**A**). Relative protein levels of IκBα (**B**), phosphorylated NFκB p65 (**C**), phosphorylated TAK1 (**D**), and phosphorylated IKKα/β (**E**). All values represent the mean ± SEM; *n* = 3. * The values are significantly different compared with the corresponding control value at *p* < 0.05, as assessed by Student’s *t*-tests.

NFκB is a crucial transcriptional regulator for the upregulation of proinflammatory cytokine mRNA. IκB binds to NFκB in the cytoplasm and interferes with the translocation of NFκB into the nucleus. After treatment with LPS, IκB is rapidly degraded, and free NFκB is phosphorylated and translocates to the nucleus, where it stimulates the mRNA expression of its target genes. IκBα protein level was significantly higher in the GGOH-pretreated cells than in the control cells after LPS stimulation (Figure 2A,B). GGOH pretreatment significantly reduced the LPS-induced phosphorylation of NFκB p65 (Figure 2A,C). The upstream kinases, TAK1 and IKK, were sequentially activated by LPS stimulation, leading to IκB degradation and nuclear translocation of NFκB. The phosphorylation of TAK1 and IKK was significantly lower in GGOH-treated cells than in control cells after LPS stimulation (Figure 2A,D,E). These results indicated that GGOH treatment interfered with NFκB activation by LPS and then suppressed inflammatory cytokine expression.

### 2.3. GGOH Suppresses IRAK1 and TRAF6 Expression and the Subsequent Phosphorylation of NFκB Signaling Molecules

**Figure 3:**
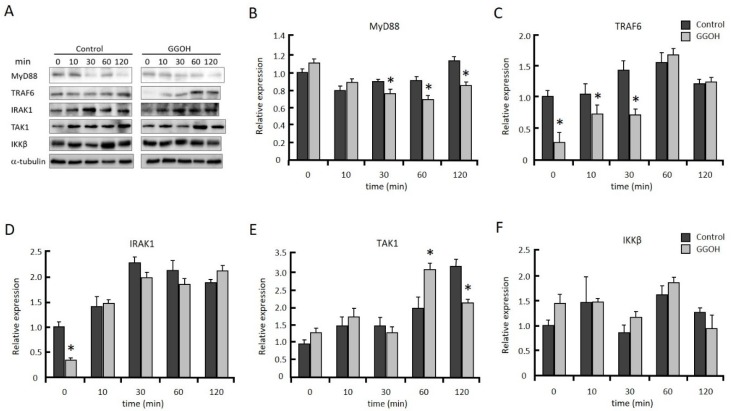
GGOH modulates NFκB signaling molecules. Differentiated THP-1 cells were incubated with or without 10 µM GGOH for 24 h, then stimulated with LPS. The cells were harvested after the indicated time of LPS treatment, and protein levels were measured by western blot, as described in the experimental procedures (**A**). Relative protein levels of MyD88 (**B**), tumor necrosis factor receptor-associated factor 6 (TRAF6) (**C**), interleukin-1 receptor-associated kinase 1 (IRAK1) (**D**), TAK1 **(E**), and IKKβ (**F**). All values represent the mean ± SEM; *n* = 3. * The values are significantly different compared with the corresponding control value at *p* < 0.05, as assessed by Student’s t-test.

MyD88, IRAK1, and TRAF6 work as upstream regulators in the activation of TAK1. We measured MyD88, IRAK1, and TRAF6, as well as TAK1 and IKKβ protein levels after LPS stimulation. TRAF6 protein level was significantly decreased in the GGOH-treatmed group compared with the control group from 0 to 30 min after LPS stimulation, while TAK1 and IKKβ levels were not significantly different (Figure 3). MyD88 protein level was significantly decreased in the GGOH-treated group from 30 to 120 min. IRAK1 protein level was significantly reduced by GGOH treatment only at the start of LPS stimulation (0 min). These results suggested that GGOH pretreatment decreased IRAK1 and TRAF6 protein levels at the start of LPS stimulation, which resulted in the suppression of LPS-induced inflammation.

**Figure 4:**
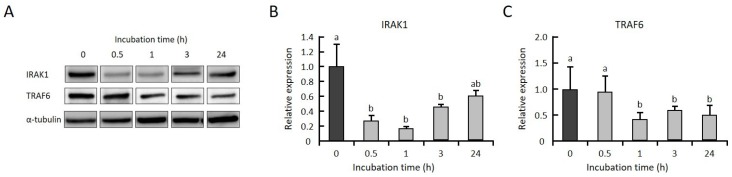

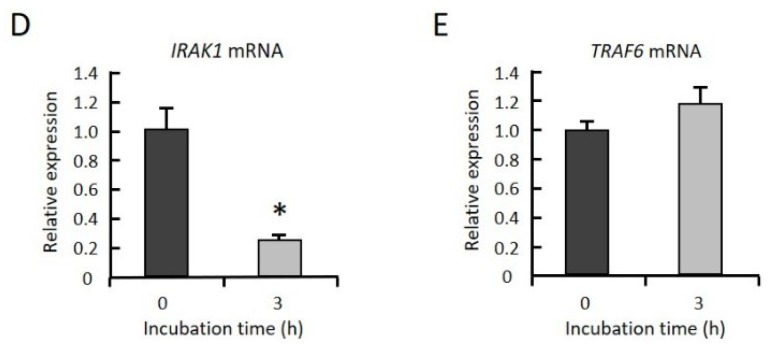
GGOH decreases the protein levels of both IRAK1 and TRAF6 but decreases the mRNA levels of only IRAK1. (**A**–**C**) Differentiated THP-1 cells were incubated with 10 µM GGOH for 0, 0.5, 1, 3, and 24 h. The cells were harvested, and the protein levels of IRAK1 (**B**) and TRAF6 (**C**) were measured by western blot, as described in the experimental procedures. All values represent the mean ± SEM; n = 3. The values with different letters (a and b) are significantly different at *p* < 0.05, as assessed by one-way analysis of variance, followed by the Fisher’s least significant difference test. (**D**,**E**) The cells were harvested, and the RNA was isolated, as described in the experimental procedures. mRNA levels of IRAK1 (**D**) and TRAF6 (**E**) were then measured by quantitative RT-PCR. All values represent the mean ± SEM; *n* = 3. * The values are significantly different compared with those at time 0 at *p* < 0.05, as assessed by Student’s *t*-test.

To clarify whether GGOH suppresses the expression levels of IRAK1 and TRAF6 prior to LPS stimulation, THP-1 cells were incubated with GGOH, and the levels of both protein and mRNA were measured. We observed that the protein levels of IRAK1 and TRAF6 were significantly decreased after 1 h of GGOH treatment (Figure 4A–C). The level of IRAK1mRNA was significantly decreased after 3 h of GGOH treatment, while the level of TRAF6 mRNA was not changed (Figure 4D,E). On the other hand, TRAF6 mRNA was significantly decreased after 24 h of GGOH treatment (Figure S1). These results indicated that the decrease in IRAK1 and TRAF6 proteins by GGOH occured in a transcriptionally dependent and independent manner at 3 h of GGOH treatment, respectively.

## 3. Discussion

**Figure 5:**
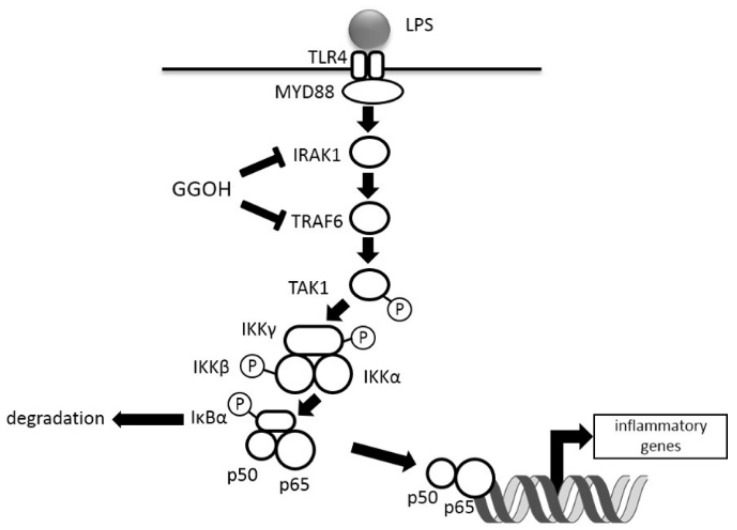
Schematic diagram of GGOH effects on LPS-stimulated NFκB activation. T arrows show the suppression of the expression levels.

We previously observed that the administration of GGOH suppressed LPS-induced inflammation in rats [14]. Here, we demonstrated an inhibitory effect of GGOH on the NFκB signaling cascade stimulated by LPS in human macrophagic THP-1 cells and showed that pretreatment with GGOH decreased the protein levels of IRAK1 and TRAF6 (Figure 5).

NFκB is a crucial transcription factor during inflammatory responses [2]. After stimulation with LPS, adaptor proteins in the NFκB signaling cascade are ubiquitinated or phosphorylated (or both) during signal transduction, beginning with the recruitment of MyD88 and IRAKs and resulting in the degradation of IκB and the nuclear translocation of NFκB [6,10,24]. NFκB binds to its response element in target genes and then stimulates the transcription of hundreds of genes, including those involved in inflammatory responses [4,24]. In this experiment, we measured the mRNA levels of proinflammatory genes (IL-1β, TNF-α, and IL-6) because these are early-response genes involved in NFκB activation downstream of TLR4 signaling. Treatment with higher GGOH doses suppressed the mRNA expression of inflammatory cytokines (Figure 1A–C), in agreement with a previous report which indicated that NFκB activation was inhibited by gavage administration of GGOH (16 mg/100 g body weight in rats) [20]. To verify the inhibition of NFκB activation, we also showed that the mRNA levels of CCL2 and COX2, additional NFκB target genes, were significantly suppressed by GGOH treatment at higher concentrations (Figure 1D,E). It is apparent that the activation of NFκB was inhibited, as shown by a decrease in phosphorylation of p65 and an increase in the abundance of IκBα (Figure 2B,C).

We did not observe a significant change in the mRNA expression levels of either TLR4 or LPS-binding protein in GGOH-treated cells. We demonstrated that treatment with GGOH suppressed IRAK1 and TRAF6 protein levels (Figure 4B,C). Similar observations of TRAF6 gene suppression and NFκB inhibition were reported in mouse macrophage RAW264.7 cells after treatment with the sesquiterpene lactone parthenolide [25]. The proteasomal degradation of TRAF6 may be attained by the polyubiquitination of Lys-48, as opposed to Lys-63, for signal transduction, and the former degradation has been reported to be promoted by the sesquiterpene lactone eupatolide [26]. In contrast, IRAK1 degradation has been reported to be initiated by rapid auto-phosphorylation because of TLR/interleukin-1 receptor (IL-1R) stimulation [27]. IRAK1 then underwent ubiquitination by its specific E3 ligase (i.e., the SCF-β-TrCP complex) [28]. However, GGOH does not bind either TLR or IL-1R. In this study, the mRNA levels of IRAK1 were significantly reduced by GGOH treatment at 3 h (Figure 4D), indicating that GGOH may have partly suppressed the protein expression of IRAK1 via transcriptional regulation. In preliminary experiments, we found the reduction of IRAK1 protein was partly mediated by the proteasome, because this IRAK1 reduction was rescued by treatment with MG132, a proteasome inhibitor. Thus, the protein levels of IRAK1 were thought to be decreased by two mechanisms, proteasome-mediated degradation and mRNA suppression. Nevertheless, as the detailed mechanism of this effect remains unclear and requires further investigation, we concluded that GGOH suppressed the protein expression levels of both IRAK1 and TRAF6.

Another possible mechanism to inhibit NFκB activation is the interference with and/or suppression of either ubiquitination or phosphorylation. It was observed that GGOH treatment substantially inhibited the phosphorylation of TAK1 and IKKα/β (Figure 2D,E), which may have been partly due to GGOH suppressing both IRAK1 and TRAF6 expression, thus inhibiting further signal transduction from TLR4. IKK consists of three distinguished proteins, IKKα, IKKβ, and IKKγ (also called NEMO, i.e., NFκB essential modulator). IKKα and IKKβ are catalytic subunits, and NEMO is an adaptor molecule that interacts with the proteins located upstream and downstream of NEMO in NFκB pathway [29]. Thus, NEMO is crucial for the regulation of IKK activity. Ubiquitinated IRAK1 (liner polyubiquitination of Met-1) interacts with NEMO in the activated NFκB pathway, and NEMO is ubiquitinated (polyubiquitination of Lys-63) by TRAF6 for its regulation [30]. Therefore, the suppression of IRAK1 and TRAF6 levels by GGOH treatment may influence the function of NEMO.

Another plausible explanation is the inhibition of IKK, NFκB-inducing kinase (NIK), or further upstream phosphorylation events by diterpenoid derivatives of geranylgeranyl pyrophosphate [31]. This hypothesis may explain why GGOH effectively inhibited signaling after 24 h of preincubation, during which time conversion to these derivatives may have occurred, and why very short incubation periods with GGOH did not inhibit the phosphorylation of IRAK1 and TAK1. We previously showed that menaquinone-4 (MK-4), a form of vitamin K_2_, inhibited the activation of NFκB in differentiated THP-1 cells by suppressing IKKα/β phosphorylation [23]. Apart from the naphthoquinone ring, MK-4 and GGOH show strikingly similar chemical structures. Other studies have also reported that γ-tocotrienol (with its multiple unsaturated isoprene units), but not γ-tocopherol, effectively inhibited NFκB activation by blocking TAK1 and other downstream signaling proteins during TNF-stimulated inflammation, without affecting DNA binding [32]. A similar inhibition of TAK1 was also observed in celastrol-treated human embryonic kidney A293 cells stimulated with TNF-α to activate NFκB [33]. We believe that more investigations on the details of how GGOH promotes degradation and inhibits phosphorylation in the NFκB signaling cascade are required. Recent studies have revealed the immunomodulatory effects of various isoprenoids and terpenoids, including GGOH, in the treatment of various diseases, providing an alternative and potential therapeutic use of natural compounds to target signal transducers.

## 4. Materials and Methods

### 4.1. Materials

GGOH was kindly provided by Tama Biochemical Co., Ltd. (Tokyo, Japan), dissolved in ethanol, and stored at −20 °C. LPS purified from *Escherichia coli* O111:B4 (Cat. #L2630), PMA, and RPMI-1640 medium were purchased from Sigma-Aldrich (St. Louis, MO, USA). Penicillin and streptomycin were purchased from Gibco Life Technologies (Carlsbad, CA, USA).

### 4.2. Cell Culture

Human monocytic THP-1 cells were obtained from the RIKEN BioResource Center (Tsukuba, Japan) and cultured in RPMI-1640 supplemented with 10% fetal bovine serum (Hyclone, Logan, UT, USA), 100 U/mL penicillin, and 100 μg/mL streptomycin at 37 °C and in a 5% CO2 atmosphere. THP-1 cells were differentiated to macrophagic cells for 48 h in culture medium containing PMA (10 ng/mL). Upon differentiation, the medium was changed with fresh medium, and the cells were used for further analyses.

### 4.3. Cell Growth Assays

Undifferentiated THP-1 cells were seeded into 96-well plates at a density of 1.2 × 104 cells/well. The medium was changed the following day to that containing PMA, and the cells were incubated for 48 h, as indicated above. Then, GGOH was added to the final concentrations of 0 and 10 µM. Following incubation for 24 h, the number of viable cells in each well was determined using the Premix WST-1 Cell Proliferation Assay System (Takara Bio Inc., Shiga, Japan) according to the manufacturer’s instructions.

### 4.4. RNA Preparation and Quantitative RT-PCR

Differentiated THP-1 cells were treated with GGOH (10 μM) for 24 h and then treated with LPS (1 μg/mL) for 3 h. After the incubation, THP-1 cells were washed with phosphate-buffered saline (PBS) twice and then homogenized in guanidine isothiocyanate-based reagent, ISOGEN (Nippon Gene, Tokyo, Japan). Total RNA was isolated from the cells according to the manufacture’s manual. RNA integrity was analyzed by agarose gel electrophoresis, and RNA quantity was determined by its absorbance at 260 nm. Five micrograms of total RNA was used for cDNA synthesis. The RNA was denatured at 65 °C with oligo-dT/random primers and 10 mM dNTP. The mixture was then incubated in 50 mM Tris-HCl buffer (pH 8.3), 0.1 mM DTT, 50 units Superscript III reverse transcriptase (Invitrogen, Carlsbad, CA, USA), and 20 units RNaseOUT RNase inhibitor (Invitrogen) at 25 °C for 5 min, at 50 °C for 60 min, and finally at 70 °C for 15 min in a TaKaRa PCR Thermal cycler MP (Takara Bio). Aliquots of the synthesized cDNA were used as a template for quantitative PCR in the Applied Biosystems 7300 Real Time PCR System (Applied Biosystems, Foster City, CA, USA). The target cDNAs were amplified by gene-specific primers (Table 1) using the SYBR Premix Ex Taq solution (Takara Bio). The expression levels of the mRNAs were normalized to the level of eukaryotic translation elongation factor 1α1 (EEF1A1) mRNA [34].

### 4.5. Western Blot Analysis

For cell samples, after a 24 h GGOH incubation (10 μM), differentiated THP-1 cells were treated with 1 μg/mL LPS for the indicated duration and then lysed at 4 °C for 30 min in ice-cold extraction buffer [35] containing proteinase inhibitors (Complete Mini proteinase inhibitor cocktail tablet, Roche Applied Science, Mannheim, Germany) and phosphatase inhibitors (PhosSTOP phosphatase inhibitor cocktail tablet, Roche Applied Science). The cell lysates were centrifuged at 15,000× *g* for 20 min, and the supernatants were collected. The protein concentration was determined using a protein assay kit (Bio-Rad Laboratories, Tokyo, Japan). Twenty micrograms of protein was denatured in SDS gel-loading buffer and electrophoresed on a 10–20% SDS–polyacrylamide gel (Wako Pure Chemical Industries, Tokyo, Japan). The proteins were then transferred onto a polyvinylidene fluoride membrane (Millipore, Billerica, MA, USA). The membrane was blocked for 1 h with TBS-T (10 mM Tris-HCl, pH 7.4, 150 mM NaCl, and 0.1% Tween20) containing 5% bovine serum albumin (Sigma) and then incubated with antibodies against IκBα (#4814, Cell Signaling Technology), MyD88 (#3699, Cell Signaling Technology, Danvers, MA, USA), IRAK1 (#4359, Cell Signaling Technology), phosphorylated IRAK1 (Thr209; ab61799, Abcam, Tokyo, Japan), TAK1 (#4505, Cell Signaling Technology), phosphorylated TAK1 (Thr184/187; #4508, Cell Signaling Technology), IKKβ (#2678, Cell Signaling Technology), phosphorylated IKKα/β (Ser176/180; #2697, Cell Signaling Technology), TRAF6 (#4743, Cell Signaling Technology), NFκB p65 (#8242, Cell Signaling Technology), or phosphorylated NFκB p65 (Ser536; #3033, Cell Signaling Technology). Bands were detected by the Immobilon Western Detection Reagent (Millipore) using the luminescent image analyzer LAS-4000 mini (Fujifilm, Tokyo, Japan). The relative expression level of each protein was normalized to the level of α-tubulin detected by an anti-α-tubulin antibody (#T9026, Sigma).

### 4.6. Statistical Analysis

Values are represented as the mean values ± SEM. One-way analysis of variance, followed by Fisher’s least significant difference test, or Student’s t-test was used to evaluate the differences between groups, unless otherwise stated. SPSS version 11.0 (SPSS Inc., Chicago, IL, USA) was used for all data analysis. Statistical significance was deemed to occur at *p* < 0.05.

## Figures and Tables

**Table 1:** Oligonucleotide sequences of primers used for quantitative RT-PCR.

Gene	Forward Primer	Reverse Primer
*CCL2*	CAAGCAGAAGTGGGTTCAGGAT	AAGTCTTCGGAGTTTGGGTTTG
*COX2*	TGAGCATCTACGGTTTGCTG	AACTGCTCATCACCCCATTC
*EEF1a1*	GATGGCCCCAAATTCTTGAAG	GGACCATGTCAACAATTGCAG
*IL-1β*	CTGATGGCCCTAAACAGATGAAGT	GCCTGAAGCCCTTGCTGTAGT
*IL-6*	ATGAGGAGACTTGCCTGGTGAA	ACTCTCAAATCTGTTCTGGAGGTACTC
*IRAK1*	CCGGGCAATTCAGTTTCTAC	TCTCATCCAGAAGGACGTTG
*TNF-α*	TGTTGTAGCAAACCCTCAAGCTG	AGGACCTGGGAGTAGATGAGGTACA
*TRAF6*	CTGCTTGATGGCATTACGAGAA	TGCAGGCTTTGCAGAACCTA

## Supplementary Materials

Supplementary Materials can be found at https://www.mdpi.com/1422-0067/20/9/2320/s1.

## Abbreviations

GGOH	Geranylgeraniol
LPS	Lipopolysaccharide
NFκB	Nuclear factor-kappa B
IRAK1	Interleukin-1 receptor-associated kinase 1
TRAF6	Tumor necrosis factor receptor-associated factor 6
