# Supplementary figures and images for: Geranylgeraniol Suppresses the Expression of IRAK1 and TRAF6 to Inhibit NFκB Activation in Lipopolysaccharide-Induced Inflammatory Responses in Human Macrophage-Like Cells
Source: Int J Mol Sci. 2019 May 10;20(9):2320. doi: 10.3390/ijms20092320 (PMC6540148; doi:10.3390/ijms20092320)

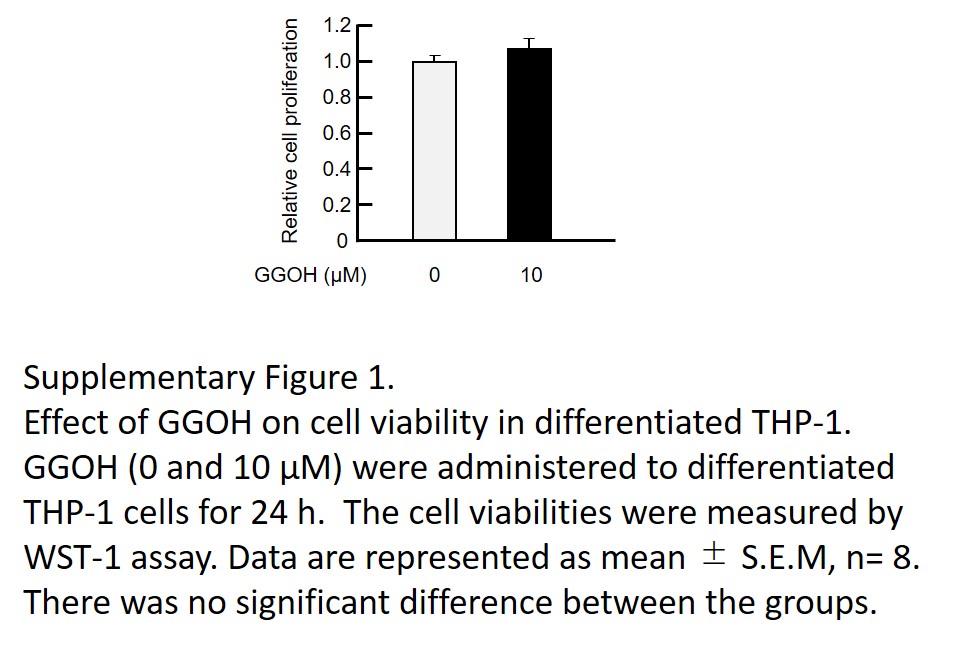

Supplement: Supplementary file 1 [file ijms-20-02320-s001.zip › supfig/SupFig1.jpg]

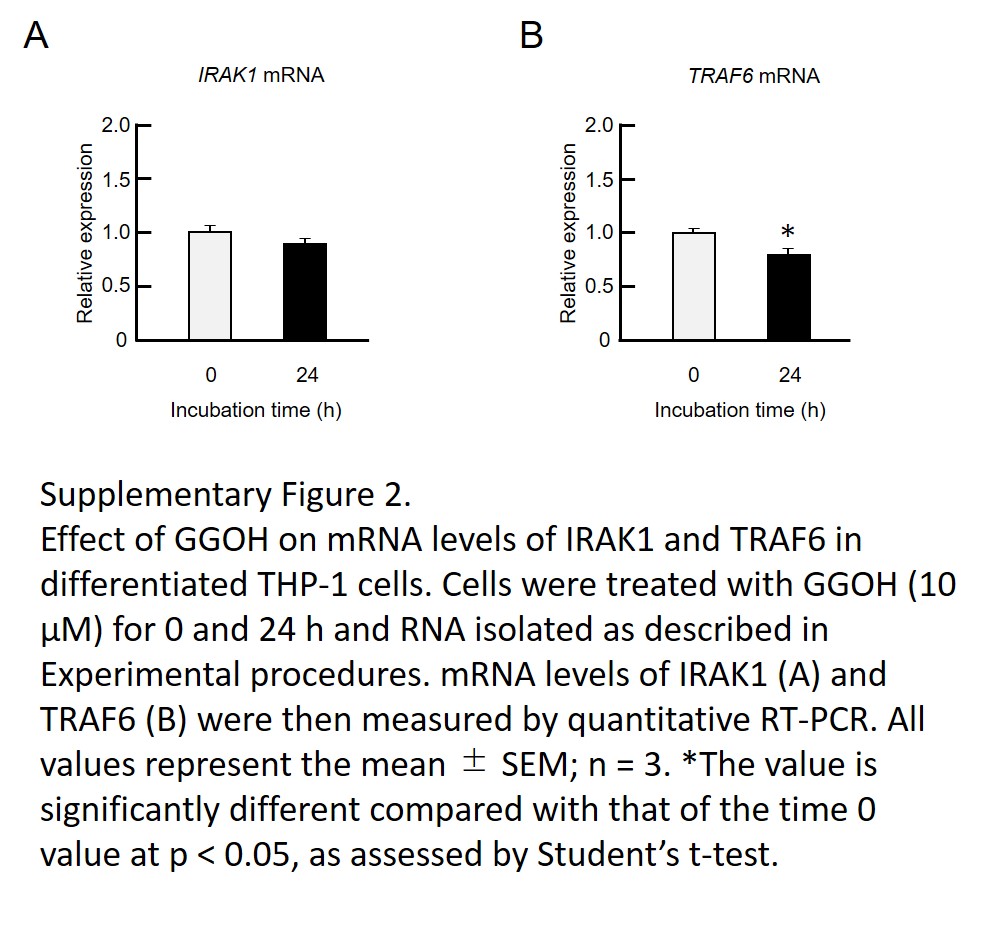

Supplement: Supplementary file 1 [file ijms-20-02320-s001.zip › supfig/SupFig2.jpg]
